# Insulin Resistance in Early Rheumatoid Arthritis Is Associated with Low Appendicular Lean Mass

**DOI:** 10.1155/2017/9584720

**Published:** 2017-08-28

**Authors:** Raili Müller, Mart Kull, Margus Lember, Kaja Põlluste, Annika Valner, Riina Kallikorm

**Affiliations:** ^1^Institute of Clinical Medicine, University of Tartu, L. Puusepa 8, 51014 Tartu, Estonia; ^2^Internal Medicine Clinic, Tartu University Hospital, L. Puusepa 8, 51014 Tartu, Estonia

## Abstract

In established rheumatoid arthritis (RA), the presence of insulin resistance (IR) is well proven but, in the early stage of the disease, data are inconclusive. We evaluated the presence of IR and associations with body composition (BC) parameters among early RA (ERA) and control subjects. The study group consisted of 92 ERA and 321 control subjects. Using homeostatic model assessment of IR (HOMA-IR), the cut-off value for IR was 2.15. 56% of the ERA patients and 25% of the controls had IR. Of the BC parameters, patients with early RA had less fat-free mass and appendicular lean mass (ALM). In multivariable model, ERA group (*b*-Coefficient) (4.8, CI: 2.6–8.8), male gender (7.7, CI: 2.7–22.1), and fat mass index (1.2, CI: 1.1–1.4) were associated with IR. Insulin-resistant ERA patients had higher inflammatory markers and higher disease activity. In the multivariable model in the ERA group, IR was associated with male gender (*b*-Coefficient) (7.4, CI: 153–34.9), high disease activity (6.2, CI: 1.7–22.2), and lower ALM (0.03, CI: 0.001–0.97). IR develops in the early stage of RA in the majority of patients. IR is more common among males and is associated with RA disease activity and lower ALM.

## 1. Introduction

Rheumatoid arthritis (RA) is a chronic systemic inflammatory disease associated with elevated cardiovascular disease (CVD) risk [1]. Cardiovascular (CV) morbidity and mortality in RA are substantially higher than among the general population already in the early stage of the disease but this excess risk is not accounted for by current methods of CV risk stratification [1–5]. RA independently increases the risk of cardiovascular disease, chiefly via two mechanisms: systemic inflammation and an excess risk of diabetes type II [2, 3]. It has been demonstrated that insulin resistance (IR), the term used to describe the inability of insulin to adequately regulate glucose metabolism in peripheral tissues, is associated with a cluster of metabolic disorders, including type 2 diabetes, obesity, hypertension, lipid abnormalities, and atherosclerotic CVD [2, 6]. Although there have been some controversial reports regarding IR and risk of atherosclerosis in RA [7, 8], IR has been associated with a 1.7-fold increase in the risk of CVD and contributes to the development of type 2 diabetes in the general population [9].

IR has been found to be significantly more frequent in established RA [2, 8]. Data on the early stage of the disease are contradictory. In a recent study evaluating IR among patients with recent onset untreated RA, no link between IR and early RA (ERA) was seen [10]. Other groups have come to the opposite conclusion, finding IR to already be more prevalent in the initial stage of the disease [11–13]. The methodological structures used in studies evaluating the presence of IR in ERA have been quite varied [10–14].

The presence of IR in RA has been suggested to be associated with seropositivity [8, 12], pro-inflammatory cytokines such as interleukin-6 (IL-6), tumor necrosis factor-alpha (TNF-alpha), disease activity, and glucocorticoid usage [2, 12, 13]. In several studies, it has been shown that anti-TNF treatment could reverse IR in RA [2, 15]. In addition to disease-specific parameters, IR in RA has been found to be associated with obesity [10, 12, 14].

RA patients are known to have higher total, truncal, and visceral fat mass than control subjects [16, 17]. Changes in body composition (BC) characterized by lean mass reduction and fat mass increase with a maintained or decreased body mass index (BMI) are common in RA [16–20]. Low lean mass and higher fat mass have been shown to already be present in the early stage of the disease [20]. The exact mechanism of sarcopenia development is unclear: TNF-alpha-driven hypermetabolism and reduced physical activity may both have potential contribution [21]. Since muscle is the primary tissue contributing to whole-body insulin-mediated glucose disposal, sarcopenia and sarcopenic obesity may be important causal factors in insulin resistance [22–24].

The aim of the study was to evaluate the presence of IR among ERA patients and control subjects, focusing on associations between IR and BC parameters.

## 2. Materials and Methods

The study group consisted of 92 patients with ERA (age: 19–79 y) and 321 subjects in the control group (age: 20–79 y). In the early RA group, 100 patients with newly diagnosed RA were recruited between 2012 and 2014. All consecutive patients referred to Tartu University Hospital with first ever rheumatoid arthritis diagnosis according to ACR/EULAR 2010 criteria for RA [25] and symptom duration up to one year (early arthritis) were invited to participate in the study. Eight patients were excluded (seven patients did not meet the ACR/EULAR criteria for RA or had another inflammatory joint disease and one patient had extremely high insulin (140.9 mU/l)) (*N* = 92).

To form the control group, 350 subjects adjusted for the age and gender composition of the Estonian population in 2013 were randomly selected from a primary health care center practice list (total number of subjects: 1854). Firstly, postal invitations with introductory materials were sent out. A total number of 332 subjects contacted the primary health care center for further instructions and were recruited during the study period. Three subjects missed their study appointment; eight with missing outcome data were excluded (*n* = 321). The age and gender distribution of the control group matched the age-sex structure of Estonian population in 2013.

Study procedures were carried out between 8 a.m. and 11 a.m. after an overnight fast. Body weight was measured in kilograms with an electronic scale, and subjects wore light indoor clothing without shoes. Height was measured to the nearest 0.5 cm using a stadiometer.

Blood samples included glucose (hexokinase assay), ESR, and CRP measurements. A panel of metabolic markers including IL-6, TNF-alpha, and insulin was evaluated using Luminex's xMAP technology. In the RA group, anti-CCP and RF levels were also evaluated. The number of tender and swollen joints was evaluated in the ERA group (28 joint scores) and DAS28 scores were calculated according to the standard formula to assess disease activity [26]. The patients were grouped according to DAS28 score as having low disease activity (DAS28 score < 3.2), moderate disease activity (≥3.2 to *≃*5.1), or high disease activity (>5.1).

BMI was calculated according to the standard formula: weight in Kg divided by height in meters squared. BMI groups were formed, normal weight (BMI: ≤24.9 Kg/m^2^), overweight (BMI: 25–29.9 Kg/m^2^), and obese (BMI: ≥30 Kg/m^2^), according to the WHO criteria [27].

BC parameters, fat mass, fat-free mass, and appendicular lean mass, were measured with a Lunar Prodigy Advance DXA machine. Fat mass index (FMI) (fat mass/height squared), fat-free mass index (FFMI) (fat-free mass/height squared), and appendicular lean mass index (ALMI) (appendicular lean mass/BMI) were calculated.

Homeostatic model assessment of IR (HOMA-IR) was calculated according to the following formula: fasting insulin (mU/L) × fasting glucose (mmol/L)/22.5 [28]. The cut-off value defined for IR was the 75th percentile of the control group values.

To evaluate the differences between the two groups, Chi-square test (Fisher's exact test when necessary) or Mann–Whitney independent-samples *U* tests were used as appropriate. Two-tailed tests and a 5% significance level were used in all analyses.

Binomial logistic regression was performed to ascertain the effects of subject characteristics on the likelihood of being classified as insulin-resistant. The linearity of the continuous variables with respect to the logit of the dependent variable was assessed via the Box-Tidwell (1962) procedure.

All analyses were carried out using SPSS v24 for Windows.

All subjects participating in the study signed written informed consent forms. The study was approved by the Research Ethics Committee of the University of Tartu.

## 3. Results

Mean age in the early RA group was 52, and it was 48 years in the control group. 72% of the patients and 54% of the controls were female. Characteristics of the study groups are presented in Table 1. Patients with ERA had significantly higher inflammatory marker values (CRP, TNF-alpha, and IL-6).

The majority of RA patients were seropositive, mean DAS28 score was 4.2 (corresponding to moderate disease activity according to ACR recommendations [26]), and mean disease duration was 215 days. Disease-specific characteristics of the ERA group are shown in Table 2.

While there was no difference between glucose values of the groups, the patients with ERA had significantly higher insulin values. Subjects with HOMA-IR > 2.15 were classified as being insulin-resistant: 55% of the ERA patients were insulin-resistant, compared to 25% of the control subjects (*p* < 0.001). Males were more likely to be classified as insulin-resistant than females, and the difference between study groups was especially profound between male patients and controls (73% versus 28%) (*p* < 0.001).

While there was no difference in BMI between the controls and ERA group, of the BC parameters, patients with ERA had lower muscle indices, less fat-free mass and appendicular lean mass. There was no difference in the measures of body fat between the groups.

Using a binomial logistic regression model, the presence of IR was independently associated with gender, RA group, FMI, and ALMI (as shown in Table 3). Early RA patients had 4.8 times higher odds of being insulin-resistant, and males had 7.7 times higher odds of exhibiting insulin resistance than females. Increasing FMI was associated with a higher likelihood of IR. The model was statistically significant (*χ*^2^ = 118.8; *p* < 0.0005). The model explained 35.3% (Nagelkerke's *R*^2^) of the variance in IR and correctly classified 74.1% of cases. Sensitivity was 44.6% and specificity was 87.9%.

Characteristics of early RA patients grouped by the presence of IR are shown in Table 4. There were proportionally more men in the insulin-resistant group. The patients classified as being insulin-resistant had higher inflammatory markers (CRP, TNF-alpha, and IL-6) and higher disease activity: mean DAS28 score in the non-IR group was 3.6, and in the IR group it was 4.6. 43% of the insulin-resistant patients had high disease activity compared to 12% of the non-insulin-resistant group. The percentage of anti-CCP and RF positivity was higher among the insulin-resistant patients but the difference did not reach statistical significance. There was no difference between the groups in fat or lean mass measures.

We conducted a binomial logistic regression for variables associated with IR among ERA patients. The logistic regression model was statistically significant (*χ*^2^ = 19.5; *p* = 0.001). The model explained 25.5% (Nagelkerke's *R*^2^) of the variance in IR and correctly classified 69.6% of cases. Sensitivity was 68.3% and specificity was 70.6%. Male ERA patients had 7.4 times higher odds of being insulin-resistant than females (as shown in Table 5). Patients with high disease activity had 6.2 times higher odds of having IR than the ones with low disease activity. Lower ALMI was associated with an increased likelihood of IR.

## 4. Discussion

In a group of 92 ERA patients and 321 subjects in the control group, patients with ERA had 4.8 times higher odds of having IR. The most important predictor for IR was male gender. Of the BC parameters, higher fat mass and lower appendicular lean mass were associated with the presence of IR. In the ERA group, high disease activity and lower appendicular lean mass index were associated with an increased likelihood of IR.

In established RA, the presence of IR is well proven, but in the early stage of the disease, the data is inconclusive, as only a handful of studies have looked into the presence of IR in ERA [2, 10–12, 14]. In this study, we tried to fill in the gap in knowledge evaluating the presence of IR in an ERA group compared to population controls. As IR is a part of a cluster of metabolic disorders and the majority of insulin-stimulated glucose disposal takes place in muscle tissue, we focused on associations between IR and BC parameters. According to results of our study, patients with recent onset RA diagnosed according to ACR/EULAR 2010 criteria [25] had significantly higher odds of presenting IR than control subjects (adjusted OR: 4.8 (CI: 2.6–8.8), *p* < 0.001, prevalence: 55% versus 25%, resp.). The difference between the groups was the most remarkable among male ERA patients and controls. The high prevalence of IR already in the initial stage of RA is of high importance, as IR in RA has been associated with both subclinical atherosclerosis and ischemic heart disease [29, 30]. While HOMA-IR is a simple and widely used measure to detect IR, there is no universal cut-off point for diagnosing IR by its value. In our study, we used the 75th percentile of the control group values corresponding to HOMA-IR 2.15. In an earlier population-based study in Estonia, the threshold value of HOMA-IR was found to be somewhat lower (1.92) [31]. Previous studies evaluating the presence of IR in ERA have differed methodologically. The rate of IR in our study is higher than the 22% that was found in a recent study conducted by Manrique-Arija et al. in a cohort of untreated ERA [10]. In a study published by Shahin et al. in 2010, the prevalence of IR was found to be 73%, reaching the conclusion that untreated RA patients are characterized by a severe insulin-resistant state [11]. The ACR 1987 criteria used to diagnose RA in this study [11, 12] have been shown to predict a more erosive disease; thus overrepresentation of patients with more severe disease is likely [32]. As several studies have come to the conclusion that IR in RA is associated with unfavorable prognostic markers of RA, this could be the reason for the unusually high prevalence of IR in this group [2, 8, 33]. Mirjafari et al. found the prevalence of IR in early inflammatory polyarthritis to be 60% by looking into Norfolk Arthritis Register data [12]. In this uncontrolled study, all patients with joint swelling of at least two joints persisting for at least four weeks were included, and only 44% of the subjects fulfilled the ACR 1987 criteria for RA. Due to their methodological differences, the studies conducted to evaluate the prevalence of IR in ERA are hard to compare.

In most of the previous studies on the topic, IR has been associated with unfavorable prognostic markers of RA: disease activity, seropositivity, and higher inflammatory activity [2]. In our study of disease-specific parameters, IR was associated with higher inflammatory burden (CRP, TNF-alpha, and IL-6) and higher disease activity. In a multivariate model, high disease activity (DAS28 score) remained statistically significant. The findings suggest that IR is tightly connected to the adverse outcome of RA and could be the reason why, in the early stage of the disease, a rise in cardiovascular mortality is already seen in seropositive patients with a higher inflammatory burden [4, 5, 34, 35]. Multiple mechanisms have been proposed by which proinflammatory cytokines promote the development of atheroma and IR. TNF-alpha induces the synthesis of SOCS3, decreases the expression of the insulin receptor substrate IRS-1 and of the glucose transporter GLUT4, and inhibits the synthesis of peroxisome proliferator-activated receptor gamma (PPAR) [2]. Additionally, it stimulates adipose tissue lipolysis, diminishes the circulating levels of adiponectin, and increases fat mass at the expense of lean mass [2]. Several studies have shown that controlling inflammation through TNF-alpha blockade in RA may improve insulin sensitivity and potentially reduce the risk of CVD in this high-risk group [2, 15]. Chronic glucocorticoid therapy is a well-known factor contributing to the development of IR; consequently, the EULAR recommends weaning patients off glucocorticoids as early as possible to avoid worsening IR and the eventual development of diabetes type II [2, 36]. The findings of our study are consistent with earlier works: at the initial stage of the disease, it does not appear to be associated with deterioration of glucose tolerance [2].

The results concerning disease-specific measures and IR are quite expected and are similar to what has been found before. In this study, we additionally evaluated BC parameters and their role in IR development. Patients with ERA had significantly lower total fat-free mass and appendicular lean mass than the subjects of the control group, with differences being most pronounced in the male groups. When looking into the combined data of the two groups, increasing FMI and decreasing ALMI were associated with a higher likelihood of IR.

We found that the lower appendicular lean mass was associated with IR in the ERA group. While several groups have shown the relationship between adipose tissue and IR in ERA [12, 14], to our knowledge, this is the first study showing increased likelihood of IR in ERA being predicted by lower muscle mass. The concept of chronic inflammation-induced muscle loss in RA is well proven in later and earlier stages of the disease [16, 17, 19–21]. Skeletal muscle accounts for the majority of whole-body insulin-stimulated glucose disposal. The inverse relationship between skeletal muscle mass and IR is a subject of wide interest and has been shown in the works of Srikanthan et al. in the general population [22, 23]. Muscle mass is an important determinant of glucose and energy homeostasis and is determined by the balance between protein synthesis and breakdown in the tissue [24]. The loss of muscle is associated with intramuscular or ectopic fat infiltration and production of adipocytokines as well as lipotoxicity, mitochondrial dysfunction, oxidative stress, IR, and anabolic resistance. In turn, these disturbances exacerbate sarcopenia, leading to a decrease in physical activity and resting energy expenditure in a self-contained loop [24, 37].

The results showed significant gender-specific differences in the presence of IR. Male gender was associated with IR; men with ERA had 7.4 times higher odds of being classified as insulin-resistant than women of the same group. Gender differences in the presence of IR in RA have not been described in works of other study groups [2, 8, 10–12, 14]. However, the association between male gender and IR has been noted in the general population and elevated visceral and hepatic adipose tissue in conjunction with the lack of a protective effect of estrogen and lower adiponectin levels are suggested to be contributing factors to the higher prevalence of IR among men compared with women [37].

There are some limitations to our study. The results showed significant gender-specific differences in the presence of IR and possibly in the factors contributing to it, and reason behind the finding is hard to explain. The sample size was insufficient for further gender-specific analysis. The patients enrolled in our study were not treatment-naïve: 55% were on DMARD treatment and 29% received corticosteroids. It is highly unlikely that this had any impact on the BC measures or presence of IR, but it may have interfered with the disease activity and inflammatory status assessment.

The results of our study emphasize the importance of routine screening for CVD risk factors starting from the initial stage of the disease. Adding an evaluation for the presence of IR could be a valuable addition to current CVD risk assessment strategies [36]. Further work is required to understand the added risk and mechanism of IR with respect to CV morbidity and mortality and possible measures for reversing and preventing the metabolic dysfunction.

## 5. Conclusion

IR already develops in the early stage of the disease in the majority of RA patients. IR is more common among males and is associated with RA disease activity and lower appendicular lean mass.

## Figures and Tables

**Table 1:** Characteristics of the study groups.

	Gender				Total	
	Male		Female			
	Early RA 26	Control group 148	Early RA 66	Control group 173	Early RA 92	Control group 321
Female, *N* (%)	—	—	—	—	**66 (71.7)**	**173 (53.9)**
Age, years	**56 (3)**	**45 (1)**	51 (2)	50 (1)	**52 (2)**	**48 (1)**
Smoking						
Ever, *N* (%)	**16 (64.0)**	**29 (19.6)**	14 (21.5)	36 (20.8)	30 (33.3)	65 (20.2)
CRP, mg/l	**21.7 (5.1)**	**1.8 (0.2)**	**7.9 (1.4)**	**2.5 (0.3)**	**11.8 (1.9)**	**2.2 (0.2)**
TNF-alpha, pg/ml	**4.0 (0.6)**	**2.1 (0.2)**	**2.3 (0.2)**	**1.8 (0.1)**	**2.8 (0.2)**	**2.0 (0.1)**
IL-6, pg/ml	**27.8 (8.3)**	**1.4 (0.9)**	**12.4 (3.7)**	**0.3 (0.1)**	**16.7 (3.6)**	**0.8 (0.4)**
Glucose, mmol/l	5.3 (0.1)	5.4 (0.1)	5.3 (0.1)	5.2 (0.1)	5.3 (0.1)	5.3 (0.0)
Insulin, mU/l	**15.3 (1.9)**	**7.2 (0.4)**	**13.2 (2.2)**	**6.9 (0.4)**	**13.8 (1.6)**	**7.0 (0.3)**
HOMA-IR score, units	**3.6 (0.4)**	**1.8 (0.1)**	**3.1 (0.5)**	**1.7 (0.1)**	**3.2 (0.4)**	**1.7 (0.1)**
Insulin-resistant, *N* (%)	**19 (73.1)**	**41 (27.7)**	**32 (48.5)**	**39 (22.5)**	**51 (55.4)**	**80 (24.9)**
BMI, Kg/m^2^	26.4 (0.9)	27.4 (0.4)	27.5 (0.8)	27.0 (0.5)	27.2 (0.6)	27.2 (0.3)
BMI group						
Normal, *N* (%)	11 (42.3)	50 (33.8)	26 (39.4)	73 (42.2)	37 (40.2)	123 (38.3)
Overweight, *N* (%)	10 (38.5)	55 (37.2)	21 (31.8)	50 (28.9)	31 (33.7)	105 (32.7)
Obese, *N* (%)	5 (19.2)	43 (29.1)	19 (28.8)	50 (28.9)	24 (26.1)	93 (29.0)
Fat mass, Kg	21.9 (1.9)	23.8 (0.9)	29.3 (1.3)	28.5 (0.9)	27.2 (1.1)	26.3 (0.6)
FMI, Kg/m^2^	7.3 (0.7)	7.4 (0.3)	11.2 (0.6)	10.6 (0.3)	10.1 (0.5)	9.1 (0.2)
FFM, Kg	**58.2 (1.2)**	**65.5 (0.7)**	**43.0 (0.7)**	**44.6 (0.4)**	**47.3 (0.9)**	**54.2 (0.7)**
FFMI, Kg/m^2^	**19.1 (0.3)**	**20.3 (0.2)**	16.2 (0.2)	16.5 (0.2)	**17.0 (0.2)**	**18.2 (0.2)**
ALM, Kg	**23.9 (0.6)**	**28.4 (0.3)**	**17.1 (0.3)**	**18.0 (0.2)**	**19.0 (0.4)**	**22.8 (0.3)**
ALMI, Kg/BMI	**0.93 (0.04)**	**1.05 (0.01)**	**0.64 (0.02)**	**0.69 (0.01)**	**0.72 (0.02)**	**0.86 (0.01)**

Values represent the mean ± SE if not stated otherwise. Statistically significant differences are shown in boldface type (*p* < 0.05). HOMA-IR: homeostatic model assessment for insulin resistance; BMI: body mass index; FMI: fat mass index (fat mass Kg/height m^2^); FFM: fat-free mass; FFMI: fat-free mass index (fat-free mass/height m^2^); ALM: appendicular lean mass; ALMI: appendicular lean mass index (appendicular lean mass Kg/BMI).

**Table 2:** RA disease-specific parameters.

	Male *N* = 26	Female *N* = 66	Total *N* = 92
Anti-CCP positive, *N* (%)	**23 (88.5)**	**40 (60.6)**	63 (68.5)
RF positive, *N* (%)	**23 (88.5)**	**41 (62.1)**	64 (69.6)
DAS28 score, units	**4.8 (0.3)**	**3.9 (0.2)**	4.2 (0.2)
Low disease activity, *N* (%)	7 (28.0)	22 (33.3)	29 (31.9)
Moderate disease activity, *N* (%)	8 (30.8)	28 (42.4)	36 (39.1)
High disease activity, *N* (%)	11 (42.3)	16 (24.2)	27 (29.73)
Disease duration, days	262.6 (57.1)	195.7 (25.3)	214.9 (24.4)
Using GCS, *N* (%)	10 (40.0)	16 (24.2)	26 (28.6)
Using DMARD, *N* (%)	11 (42.3)	40 (60.6)	51 (55.4)

Values represent mean ± SE if not stated otherwise. Statistically significant differences between male and female patients are shown in boldface type (*p* < 0.05). RF: rheumatoid factor; DAS28: disease activity score calculated using 28 joints; GCS: glucocorticosteroids; DMARD: disease-modifying antirheumatic drugs. Low disease activity (DAS28 score < 3.2), moderate disease activity (DAS28 score ≥ 3.2 to *≃*5.1), and high disease activity (DAS28 score > 5.1).

**Table 3:** The effects of age group, gender, body composition indices, and having early RA on the Likelihood of IR.

	*B *	±SE	Sig.	exp(*B*)	95% CI for exp(*B*)	
					Lower	Upper
Group	1.57	0.31	<0.001	4.80	2.61	8.82
Gender	2.04	0.54	<0.001	7.70	2.69	22.08
Age groups			NS			
Age group < 29	0.53	0.44	NS	1.69	0.71	4.0
Age group 30–49	−0.22	0.31	NS	0.80	0.44	1.47
FMI	0.20	0.06	0.002	1.21	1.08	1.38
FFMI	0.07	0.08	NS	1.08	0.92	1.27
ALMI	−3.02	1.57	0.05	0.05	0.002	1.06

*Note*. Gender is for males compared to females, early RA group compared to control group, and age groups < 29 and 30–49 compared to >50. FMI: fat mass index (fat mass Kg/height m^2^); FFMI: fat-free mass index (fat-free mass/height m^2^); ALMI: appendicular lean mass index (appendicular lean mass Kg/BMI).

**Table 4:** Characteristics of non-insulin-resistant and insulin-resistant early RA patients.

	Insulin resistance		*p* value
	Non-insulin-resistant *N* = 41	Insulin-resistant *N* = 51	
Age, years	51 (2)	54 (2)	NS
Gender			
Male, *N* (%)	7 (17.1)	19 (37.3)	0.02
BMI, Kg/m^2^	26.3 (0.8)	27.9 (0.9)	NS
CRP, mg/l	8.6 (3.0)	14.5 (2.4)	0.01
TNF-alpha, pg/ml	2.1 (0.1)	3.4 (0.4)	0.001
IL-6, pg/ml	8.6 (4.2)	23.2 (5.3)	<0.001
RF positive, *N* (%)	25 (61.0)	39 (76.5)	NS
Anti-CCP positive, *N* (%)	25 (61.0)	38 (74.5)	NS
DAS28 score, units	3.6 (0.2)	4.6 (0.2)	0.003
Low disease activity, *N* (%)	18 (45.0)	11 (21.6)	0.02
Moderate disease activity, *N* (%)	18 (43.9)	18 (35.3)	NS
High disease activity, *N* (%)	5 (12.2)	22 (43.1)	0.001
GCS usage			
Current user, *N* (%)	10 (25.0)	16 (31.4)	NS
DMARD usage			
Current user, *N* (%)	24 (58.5)	27 (52.9)	NS
Disease duration, days	234.7 (37.5)	199.5 (32.3)	NS
Total fat mass, Kg	26.2 (1.7)	28.1 (1.6)	NS
FMI, Kg/m^2^	9.5 (0.6)	10.6 (0.7)	NS
FFM, Kg	46.4 (1.2)	48.0 (1.4)	NS
FFMI, Kg/m^2^	16.6 (0.3)	17.4 (0.3)	NS
ALM, Kg	18.7 (0.6)	19.3 (0.6)	NS
ALMI, Kg/BMI	0.73 (0.03)	0.72 (0.03)	NS

Values represent mean ± SE if not stated otherwise. BMI: body mass index; CRP: C-reactive protein; TNF-alpha: tumor necrosis factor-alpha; IL-6: interleukin 6; RF: rheumatoid factor; DAS28: disease activity score calculated using 28 joints; GCS: glucocorticosteroid; FMI: fat mass index (fat mass Kg/height m^2^); FFM: fat-free mass; FFMI: fat-free mass index (fat free mass/height m^2^); ALM: appendicular lean mass; ALMI: appendicular lean mass index (appendicular lean Kg/BMI).

**Table 5:** Summary of binary logistic regression analysis for variables predicting insulin resistance in the ERA Group.

	*B*	±SE	Sig.	exp(*B*)	95% CI for exp(*B*)	
					Lower	Upper
Gender	2.0	0.80	0.01	7.35	1.55	34.91
ALMI	−3.45	1.74	0.05	0.03	0.001	0.97
TNF-alpha (pg/ml)	0.42	0.26	NS	1.52	0.91	2.53
DAS28 score			NS			
High DAS28 score	1.82	0.65	0.005	6.19	1.72	22.2
Moderate DAS28 score	0.48	0.53	NS	1.62	0.57	4.59

*Note*. Gender is for males compared to females. ALMI: appendicular lean mass index (appendicular lean Kg/BMI); TNF-alpha: tumor necrosis factor-alpha; DAS28: disease activity score calculated using 28 joints. High and moderate DAS28 score compared to low score.
